# Selected Factors of Innate Immunity in Healthy Individuals with *S. aureus* Nasal Carriage

**DOI:** 10.3389/fmicb.2016.00453

**Published:** 2016-03-31

**Authors:** Tomasz M. Karpiński, Zbigniew Żaba, Izabela Chudzicka-Strugała, Anna K. Szkaradkiewicz, Agata Jaworska, Agnieszka Zeidler, Ewa Andrzejewska, Andrzej Szkaradkiewicz

**Affiliations:** ^1^Department of Medical Microbiology, Poznan University of Medical SciencesPoznań, Poland; ^2^Department of Teaching Anaesthesiology and Intensive Therapy, Poznan University of Medical SciencesPoznań, Poland; ^3^Department of Conservative Dentistry and Periodontology, Poznan University of Medical SciencesPoznań, Poland

**Keywords:** *Staphylococcus aureus*, nasal carriage, human beta-defensin-2 (HBD-2), lysozyme (Ly), interferon-gamma (IFN-γ)

## Abstract

Nasal carriage of *Staphylococcus aureus* represents a well-defined factor of risk involving community and hospital-acquired infections. Recently a significance of several host factors has been pointed out and, in particular, of immune determinants in nasal *S. aureus* colonization. Therefore, this study aimed at analysis of manifestation involving manifestation in the nasal secretions of important components of the host innate immunity – human beta-defensin-2 (HBD-2), lysozyme (Ly), and interferon-gamma (IFN-γ) in healthy individuals and in persons with persistent carriage of *S. aureus*. The studies were conducted in two groups of healthy volunteers, encompassing non-carriers (group 1) or persistent carriers of *S. aureus* (group 2). Elisa assays were employed to evaluate levels of HBD-2, Ly, and IFN-γ in nasal secretions of the examined donors. In *S. aureus* carriers a significant variability of HBD-2 levels was detected, corresponding to, respectively, the high (averaging at 1.46 ng/ml) and the low (averaging at 0.13 ng/ml) secretory response of the defensin. The level of Ly in *S. aureus* carriers averaged at 1.46 μg/ml and it manifested no significant difference as compared to that noted in non-carriers. In turn, concentrations of IFN-γ in nasal secretions in the group of carriers of *S. aureus* amounted on the average to 81.7 pg/ml and they were 1.3-fold higher that in the group of non-carriers. The obtained results allow to conclude that IFN-γ secretion by the nasal cavity-colonizing *S. aureus* remains quantitatively insufficient to eliminate the pathogen. Nevertheless, a significant increase in levels of this host factor may be important for restriction of the staphylococcal colonization and protection against development of an invasive infection. In turn, the role of HBD-2 and Ly in inactivation of the colonizing *S. aureus* remains doubtful.

## Introduction

*Staphylococcus aureus* represents one of the most frequently occurring community and hospital-acquired pathogens (Wertheim et al., 2004; Verhoeven et al., 2014). The vestibulum nasi is the primary reservoir of *S. aureus* in humans, and nasal carriage has been related to an increased risk of staphylococcal disease (Wertheim et al., 2005; Verhoeven et al., 2014). The nasal strains *S. aureus* originating from carriers were demonstrated also to carry toxin genes, most frequently the gene coding for toxic shock syndrome toxin 1 (TSST-1) – *tst* (Mehrotra et al., 2000). Moreover, persistent carriers exhibited higher than in non-carriers serum anti-staphylococcal antibodies targeted at TSST-1 (Verkaik et al., 2009). At present, two categories of nasal carriers are distinguished, involving persistent and non-persistent carries, respectively (Van Belkum et al., 2009). The persistent nasal carriage of *S. aureus* is appraised to affect around 20–30% of the whole population, in Poland on the average 28% of healthy adults (Wertheim et al., 2005; Chudzicka-Strugała et al., 2015). In the process of *S. aureus* nasal colonization various bacterial factors play role, mainly adhesion molecules such as surface components, including clumping factor B (ClfB) and cell wall teichoic acids (Mulcahy et al., 2012; Weidenmaier et al., 2012). Nevertheless, genetic investigations indicate that there exists no specific bacterial factor linked to persistent nasal carriage of *S. aureus* (Lamers et al., 2011). However, a significance of certain bacterial species in the normal flora was pointed out for reduction of *S. aureus* nasal colonization (Frank et al., 2010). In parallel, involvement of host innate immunity, in particular of defensins and of the already well known antibacterial peptides was accepted in reduction of *S. aureus* nasal carriage (Van Belkum et al., 2007; Sollid et al., 2014). Nevertheless, the role of this defensin and of the other important determinants of innate immunity still remains unclear in the nasal *S. aureus* colonization.

Therefore, this study aimed at analysis of the presence in the nasal secretions of human beta-defensin-2 (HBD-2), lysozyme (Ly), and interferon-gamma (IFN-γ) in healthy individuals and in persons with persistent nasal carriage of *S. aureus*.

## Materials and Methods

### Patients

The studies were performed in the Department of Medical Microbiology, Poznań University of Medical Sciences, over a period of 2 years (2014–2015). All the research protocols were reviewed and approved by the Ethics Committee at the Poznań University of Medical Sciences, Poland. All subjects gave written informed consent in accordance with the Declaration of Helsinki. Sixty persons were qualified for the studies, in two research groups. The first group (group 1) included 30 persons (20 womens and 10 mens), 19–24 years of age, classified as non-carriers of *S. aureus*. Group 2 comprised 30 persons (18 womens and 12 mens), 20–24 years of age, confirmed persistent *S. aureus* nasal carriers. For the carriers, nasal cultures were positive for *S. aureus* on two occasions during a minimum interval of 3 months, according to Panierakis et al. (2009). The volunteers did not include health care workers. The investigated groups included no individuals with current infection or reporting chronic diseases or persons reporting in anamnesis genetic diseases. Moreover, persons with anatomic alterations in the nose, smokers, individuals administered with oral contraceptives and i.v., drug users were excluded. Within the previous 2 weeks, none of the patients in the mentioned groups was administered with antibiotics/chemotherapeutic agents locally or systemically. Additionally, dental examinations of patients failed to find any potential infection foci in the oral cavity. The investigated material involved nasal swab samples for detection of bacterial carriage and nasal secretions for estimation of levels of HBD-2, Ly, and IFN-γ. Nasal secretions were collected by vacuum-aided suction without chemical stimulation, as earlier described (Cole et al., 1999). Gentle manipulation of a rubber-tipped vacuum device inside the nasal passageways stimulated release of nasal fluid. Nasal secretions were stored at –20°C until further analyses were performed.

### Detection of *S. aureus*

The bacteria were isolated on sheep blood agar within 20–24 h at a temperature of 37°C in aerobic conditions. The developed colonies were subsequently identified using conventional techniques (colony morphology, evaluation of haemolysis, staining according to Gram, production of coagulase, catalase, ability to decompose mannitol in Chapman medium). Identification of *S. aureus* was conducted using the automated system ATB with the application of ID 32 Staph strips (bioMérieux). Moreover, presence of *S. aureus* was confirmed using PCR. All strains of *S. aureus* proved to be methicillin-sensitive (MSSA).

### Detection of *S. aureus* using PCR

DNA was isolated from the obtained isolates of *S. aureus* clinical strains. At first, the samples were digested with lysostaphin (10 μl of 1 mg/ml solution) and incubating them for 10 min at a temperature of 37°C. Subsequently, for the isolation of DNA, Swab kits (A&A Biotechnology) were used. The isolation of DNA was conducted as recommended by the manufacturer. The purified DNA was stored at –20°C until further analyses were performed. In PCR studies the following oligonucleotide primers were used according to Martín-López et al. (2004): femB1 5′-TTACAGAGTTAACTGTTACC-3′ and femB2 5′-ATACAAATCCAGCACGCTCT-3′ from *femB* gene of *S. aureus*. PCR reaction was conducted in 25 μl of mixture consisting of 1x reaction buffer [10 mM Tris-HCl (pH 8.3)], 2.5 mM MgCl_2_, 0.2 mM of each of the four dNTPs, 1.25 U of Taq DNA polymerase, 0.2 μM of each primer and 1 μl of templete DNA. The PCR reaction was conducted in the Mastercycler Pro S thermocycler (Eppendorf) with the following thermal cycling profile: an initial denaturation at 94°C for 4 min, denaturation at 94°C for 45 s, annealing at 50°C for 45 s, and extension at 72°C for 60 s, ending with a final extension step at 72°C for 10 min. The number of cycles in PCR reaction was 30. The PCR product was subjected to electrophoresis in 1% agarose gel and the result was recorded following staining with ethidine bromide. A positive result was accepted to involve presence of PCR reaction product of 651 bp in size.

### Elisa Tests

Levels of HBD-2, Ly, and IFN-γ in nasal secretions were estimated by Elisa technique using kits of Human Beta Defensin 2 (Alpha Diagnostic), Lysozyme Elisa Kit (Sigma), and Human IFN-γ High Sensitivity Elisa (eBioscience). Sensitivity of the applied tests amounted to 5 pg/ml, 0.021 ng/ml and 0.06 pg/ml, respectively. The tests were performed as recommended by the manufacturers. Values of absorbance, depending on the estimated substance, were read using Reader 250 (bioMérieux). The results were obtained from standard curves. Every test was performed three times and the presented result involved mean of the estimations.

### Data Analysis

Results obtained in the studies were subjected to statistical analysis employing the computer Statistica 8 software for the Windows operational system. In comparative analysis of studied factors in the groups, the nonparametric test of Mann–Whitney and Kruskal–Wallis with Dunn’s was employed. The relationships with *P*-values higher than 0.05 were considered insignificant.

## Results

**FIGURE 1:**
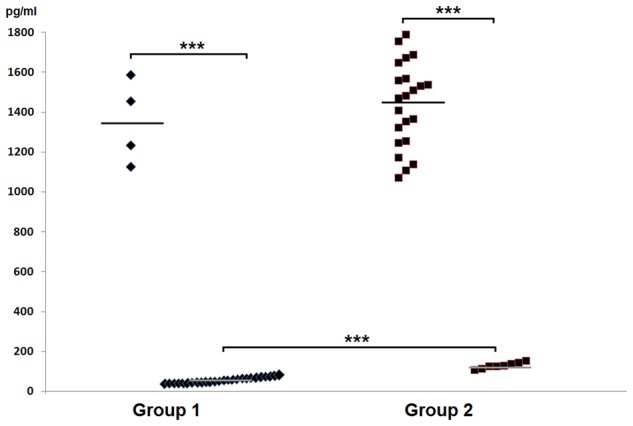
**Graphic presentation of detected levels of HBD-2 in the nasal secretions in non-carriers *Staphylococcus aureus* (group 1) and in carriers of *S. aureus* (group 2)**.

In the group 1 of healthy volunteers (non-carriers *S. aureus*) in 26 (87%) persons mean level of HBD-2 amounted to 68.42 ± 18.06 pg/ml, while in 4 (13%) persons the level was significantly elevated, amounting to 1352.75 ± 206.47 pg/ml. In group 2 two categories of results were obtained: in 22 (73%) carriers of *S. aureus* the mean level of HBD-2 was very high and it amounted to 1456.23 ± 202.68 pg/ml, while in 8 (27%) persons concentrations of the defensin were low, averaging at 132.13 ± 15.82 pg/ml. In parallel, the results proved to be statistically higher than those detected in 87% non-carriers. The obtained results were summed up in **Table 1** and, graphically, in **Figure 1**.

**Table 1:** Levels of HBD-2 in the nasal secretions in non-carriers *Staphylococcus aureus* (group 1) and in carriers of *S. aureus* (group 2).

Studied defensin	Mean values ± SD (minimun–maximum)		*P* between groups
		
	group 1 *n* = 30	group 2 *n* = 30	
HBD-2 (pg/ml)	*n* = 26	*n* = 8	<0.0001
	68.42 ± 18.06	132.13 ± 15.82	
	(32.2–103.8)	(108.7–156.4)	
	*n* = 4	*n* = 22	>0.05
	1352.75 ± 206.47	1456.23 ± 202.68	
	(1131–1587)	(1121–1775)	


**FIGURE 2:**
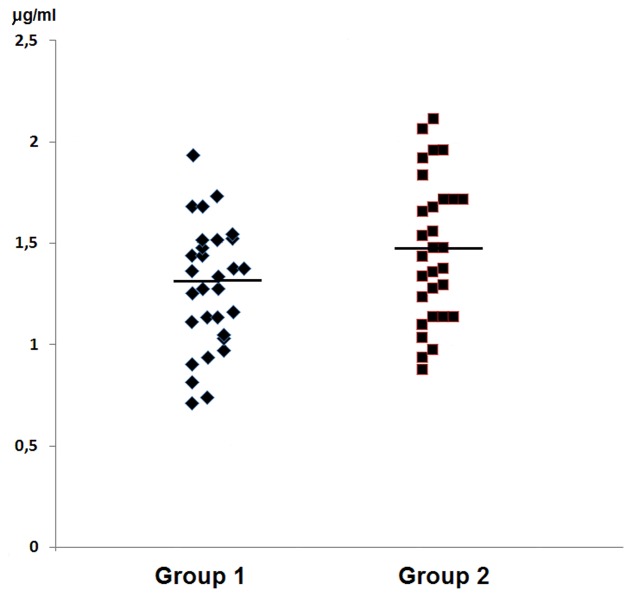
**Graphic presentation of detected levels of Ly in the nasal secretions in non-carriers *S. aureus* (group 1) and in carriers of *S. aureus* (group 2)**.

In group 1 of healthy volunteers (non-carriers *S. aureus*) the mean level of Ly amounted to 1.46 ± 0.34 μg/ml, while in group 2 of *S. aureus* carriers the mean level amounted to 1.29 ± 0.30 μg/ml. Levels of the enzyme showed no significant inter-group difference; they were presented in **Table 2** and in **Figure 2**.

**Table 2:** Levels of Ly and IFN-γ in the nasal secretions in non-carriers *S. aureus* (group 1) and in carriers of *S. aureus* (group 2).

Studied factor	Mean values ± SD (minimum–maximim)		*P* between groups
		
	group 1 *n* = 30	group 2 *n* = 30	
Ly (μg/ml)	1.29 ± 0.30	1.46 ± 0.34	>0.05
	(0.72–1.94)	(0.88–2.12)	
IFN-γ (pg/ml)	63.8 ± 14.2	81.7 ± 14.8	<0.0001
	(42.3–97.6)	(50.7–112.2)	


**FIGURE 3:**
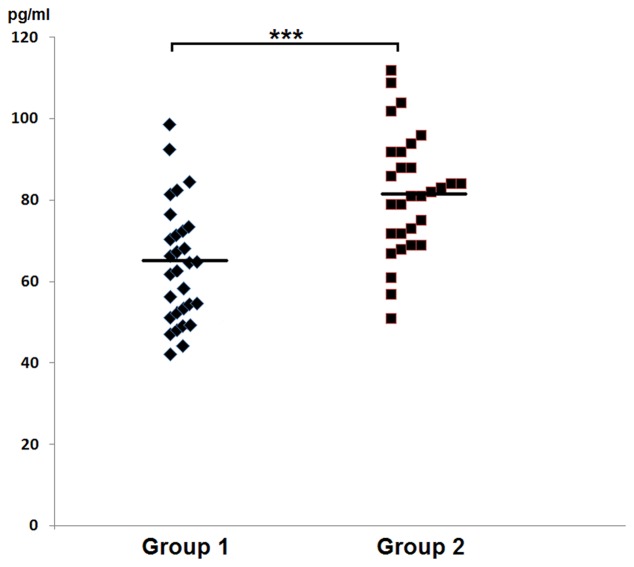
**Graphic presentation of detected levels of IFN-γ in the nasal secretions in non-carriers *S. aureus* (group 1) and in carriers of *S. aureus* (group 2)**.

In group 1 of healthy volunteers (non-carriers of *S. aureus*) the mean level of IFN-γ was 63.8 ± 14.2 pg/ml, while in the group 2 of *S. aureus* carriers it amounted to 81.7 ± 14.8 pg/ml. In the latter group levels of IFN-γ proved to be significantly higher than those in group 1 of non-carriers. The results were summed up in **Table 2** and in **Figure 3**.

## Discussion

Several host factors which spontaneously exert anti-microbial activity create innate immunity against infections (Boman, 2000; Brown et al., 2014). In this study we conducted investigations related to presence of three important determinants of innate immunity, HBD-2, Ly, and IFN-γ in nasal fluids of healthy persistent carriers of *S. aureus* and in non-carriers.

HBD-2 involves an antimicrobial peptide, produced mainly by skin keratinocytes and respiratory epithelial cells in response to infection and inflammation (Schneider et al., 2005). Currently, the peptide is thought to form the first line of local defense at the mucosal surface (Guaní-Guerra et al., 2010). In analysis of HBD-2 manifestation in nasal fluids originating from the examined volunteers we obtained two categories of results both in the group of carriers of *S. aureus*, and among non-carriers. In the latter, in around 87% individuals we detected low levels of the defensin, not exceeding 0.1 ng/ml, while in around 13% volunteers level of the peptide in nasal fluids was high, exceeding 1 ng/ml. The high levels of HBD-2 were detected also in around 73% carriers of *S. aureus*. On the other hand, in around 27% of the remaining carriers levels of the defensin remained within low concentrations even if they were significantly higher than those detected in 87% non-carriers. The data correspond with those obtained by Cole et al. (2001), who documented high concentrations of HBD-2 in nasal fluids of persistently colonized carriers. However, the quoted authors failed to provide the number of donors in whose nasal fluids the peptide was analyzed and the employed by them ELISA test showed low sensitivity (0.125 ng/ml). Therefore, results of their paper can only partially be compared to our results. The detected by us in persistent *S. aureus* carriers significant variability in levels of HBD-2, may represent a result of the presence among healthy individuals of “high responders” and “low responders,” depending on the level of induction involving expression of HBD-2 genes in nasal epithelial cells. Such a conclusion seems to be probable in view of results obtained by Nurjadi et al. (2013), who demonstrated associations between genetic polymorphisms, β-defensin (HBD-1 and HBD-3) expression, and persistent *S. aureus* nasal carriage. A suppressive effect is also possible, exerted in the carriers by *S. aureus* strains on expression of the defensin, as suggested by Quinn and Cole (2007). The presented data allow to assume that the action of HBD-2 against the colonizing *S. aureus* remains ineffective. In contrast to this suggestion seem to argue studies of Routsias et al. (2010), who demonstrated *in vitro* high bactericidal efficacy of the defensin against *S. aureus* clinical strains. Significance of the studies, however, was reduced by interpretation because the quoted authors used in their experiments HBD-2 concentrations significantly higher than the defensin levels detected by us in the nasal fluids. Moreover, studies on healthy skin specimens originating from patients with persistent nasal carriage of *S. aureus* and from non-carriers, conducted by Zanger et al. (2011) indicated that nasal colonization by the pathogen was promoted by a deficit in HBD-3. Thus, the significantly elevated concentrations of HBD-2 in nasal secretions of carriers may represent just a sequel of local inflammatory response against colonizing *S. aureus*. In turn, the high levels of the defensin detected in this study in some non-carriers may reflect its induction by transient nasal microflora, and the Gram-negative bacilli in particular. Stimulation of HBD-2 expression by bacterial LPS is already well known (Guaní-Guerra et al., 2010).

Lysozyme represents a polypeptide manifesting enzymatic activity (muramidase activity), hydrolysing β-1,4 glycosidic bonds between N-acetylmuramic acid and N-acetylglucosamine of bacterial peptidoglycan (Dziarski, 2004). The enzyme creates an important component of the host innate immunity. It is present ubiquitously in various human tissues and secretions, exhibiting antimicrobial activities against different microorganisms (Dumoulin et al., 2007). Ly was first reported in nasal secretions by Fleming (1922). In our study we have found that nasal fluids of healthy donors contain Ly levels within the range of 0.7–1.9 μg/ml, manifesting no significant difference as compared to nasal fluids in carriers of *S. aureus*. It is difficult to compare the results to those obtained in earlier studies (Raphael et al., 1989; Noble, 2002), the authors of which estimated Ly activity using a turbidimetric assay, based on the enzymatic hydrolysis of bacterial cell walls. Therefore, the obtained data allow to conclude that Ly does not prevent against nasal carriage of *S. aureus*. The conclusion is supported by studies pointing to high resistance of peptidoglycan of *S. aureus* to lytic activity of Ly (Pushkaran et al., 2015). However, a non-lytic mechanism of the enzyme activity is also known, related to its cationic and hydrophobic properties, which results in bacterial autolysis (Masschalck et al., 2002). Chen et al. (2005) in their *in vitro* analysis of Ly action demonstrated its antibacterial activity toward *S. aureus* and, moreover, demonstrated a synergism of Ly and HBD-2 actions. However, the phenomenon manifested statistically significant effects only in an acidic milieu (pH 4.6), while nasal fluids manifest a neutral or slightly alkaline pH (Lee et al., 2009). In the context of the data it can be concluded that Ly concentrations in nasal fluid are not sufficient to secure effective elimination of the colonizing *S. aureus*.

IFN-γ represents a typical cytokine, produced mainly by lymphocytes T and NK (Young and Hardy, 1995). The cytokine plays a central role in the innate immunity to infection. It also exerts a strong monocyte/macrophage-stimulating effect, activating phagocytosis and mechanisms for intracellular killing of pathogens (Schroder et al., 2004). In this study, using high sensitivity Elisa test for the first time we have documented IFN-γ estimations in nasal fluids in carriers of *S. aureus*. The detected by us levels of the cytokine proved to be comparable to results recently obtained by König et al. (2015), using a commercial immunofluorescence multiplexed assay. Also, the concentrations of IFN-γ detected in nasal secretions of *S. aureus* carriers were on the average 1.3-fold higher that those present in non-carriers. The results correspond to those obtained in the recently published studies of Brown et al. (2015) on a mouse *in vitro* model and *in vitro* human estimations which showed that *S. aureus* enhanced IFN-γ response, which might be followed by an augmented microbicidal activity of phagocytes and by elimination of the pathogens. In this context, persistence of *S. aureus* carriage may be linked to an insufficient increase of IFN-γ level to clear the colonizing pathogen. Moreover, a defect in phagocyte function is also possible, even if in anamnesis the examined individuals provided no respective indications. However, the elevated level of IFN-γ may at least in part mediate a microbicidal activity, preventing against spread of the infection.

## Conclusion

The presented data indicate that the detected by us induction of IFN-γ secretion by nasal cavity-colonizing *S. aureus* remained quantitatively insufficient to eliminate the pathogen. Nevertheless, the significant increase in the host factors may be of importance for a reduced staphylococcal colonization and protection against spread of the invasive infection. On the other hand, involvement of HBD-2 and Ly in inactivation of the colonizing *S. aureus* seems doubtful.

## Author Contributions

Conceived and designed the experiments: TK and AS. Material collection: ZŻ, IC-S, and EA. Performed the experiments: TK, AS, AJ, and AZ. Analyzed the data: TK, AS, and AS. Wrote the manuscript: TK and AS.

## Conflict of Interest Statement

The authors declare that the research was conducted in the absence of any commercial or financial relationships that could be construed as a potential conflict of interest.
